# Palliative care of older glioblastoma patients in neurosurgery

**DOI:** 10.1007/s11060-022-03985-x

**Published:** 2022-03-24

**Authors:** Daniel Berthold, Anna Pedrosa Carrasco, Eberhard Uhl, Heidi Müller, Rio Dumitrascu, Ulf Sibelius, Holger Hauch

**Affiliations:** 1 Clinic for Internal Oncology, Haematology and Palliative Care, University Hospital of Giessen and Marburg, Giessen Site, Giessen, Germany; 2 Research Group Medical Ethics, Faculty of Medicine, Philipps University Marburg, Marburg, Germany; 3 Department of Neurosurgery, University Hospital of Giessen and Marburg, Giessen Site, Giessen, Germany; 4 Palliative Care Team, University Children’s Hospital, University Hospital of Giessen and Marburg, Giessen Site, Giessen, Germany

**Keywords:** Palliative care, Geriatric neurosurgery, Glioblastoma, Older patients, End-of-life

## Abstract

**Purpose:**

The care of older neurosurgical patients at the end life is a particularly demanding challenge. Especially, the specific needs of very old patients with glioblastoma at the end of life are at risk of being deprived of adequate care.

**Methods:**

Based on a narrative literature review, this article aims to explore key issues of the thematic intersection of geriatric glioblastoma patients, palliative care and neurosurgery.

**Results and discussion:**

Four key issues were identified: patient-centeredness (need orientation and decision making), early palliative care, advance care planning, and multi-professionalism. Possible benefits and barriers are highlighted with regard to integrating these concepts into neurosurgery.

**Conclusions:**

Palliative care complements neurosurgical care of geriatric glioblastoma multiforme patients to optimise care for this highly vulnerable category of patients.

## Introduction

According to the WHO definition, “Palliative care is an approach that improves the quality of life of patients (adults and children) and their families who are facing problems associated with life-threatening illness. It prevents and relieves suffering through the early identification, correct assessment and treatment of pain and other problems, whether physical, psychosocial or spiritual” [1]. Palliative questions in neurosurgical patients can come to the fore when patients suffer from progressive neurological diseases, but also when nonneurological underlying diseases cause neurological complications. Progressive neurological diseases include for example degenerative diseases (e.g., Parkinson’s disease), infectious diseases that lead to increased intracranial pressure (e.g., encephalitides) or primary tumours of the central nervous system (e.g., glioblastoma multiforme). Nonneurological underlying diseases include metastases of tumours that do not primarily arise from the nervous system, but cause neurological symptoms and deficits (e.g., brain metastases due to primary lung and breast carcinomas).

The care of neurosurgical patients at the end of life, especially of those suffering from glioblastoma multiforme (GBM), is a particularly demanding challenge for two reasons. First, it is often difficult to predict the course of GBM requiring neurosurgical intervention, especially as GBM is considered a model disease of a rapidly progressive malignant tumour in the neurosurgical context [2]. It is therefore difficult to estimate the start of the terminal or final phase. Patients may get better (once again) and enter a phase of renewed stabilisation. Particularly in GBM patients, there are frequent cases in which new acute neurological deficits require a quick and unprepared decision with significant consequences, including whether potentially life-saving but risky neurosurgical intervention should be performed. There is often no time left for the careful elaboration of palliative treatment concepts, including the (presumed) will of the patient, the relatives’ perspective and ethical considerations.

Second, the incidence of GBM peaks between 65 and 84 years of age [3]. Hence, the challenge is due to the high complexity of the illness situation in many older individuals, so treatment options might be impeded by an already complex geriatric disease history.

In the upcoming years, increasing life expectancy may result in an increasing number of neurosurgical patients suffering from brain tumours. Older patients in particular are at risk of being deprived of adequate end-of-life care. Studies indicate that older people have less access to palliative care structures [4, 5]. In addition, Hunt et al. [6] demonstrated that patients aged 85 years and older are comparatively poorly cared for during the last two days of their lives, both in terms of nonpain symptoms, as well as emotionally and spiritually. Reasons for the low referral rates of geriatric patients from referrer perspective include the persistent focus on curative medicine, a higher level of acceptance of terminal diagnoses among older patients, the lack of pressure from family environment, and the lack of awareness for considering palliative care for nononcological patients [7]. Specific needs of the very old with GBM at the end of life may run the risk of being not properly addressed. Against this backdrop, we consider it worthwhile to investigate topical overlaps in the care of GBM patients in geriatrics, palliative care and neurosurgery. Based on a narrative review, this article aimed to identify key issues of the thematic intersection of these factors. Furthermore, barriers and facilitators are addressed that exist at the border between neurosurgery and palliative care to sensitise service providers and ultimately optimise patient care.

## Methods

A narrative literature review was conducted. In contrast to systematic reviews, narrative reviews have the aim of increasing the understanding of a larger field by summarising, explaining and interpreting qualitative and quantitative findings [8]. Nevertheless, our research is prone to bias as a result of streamlining the systematic review process requiring cautious interpretation of results. However, it has the advantage of being able to explore a wide range of current issues without too strict methodological limitations. Thorough and structural analyses of this review’s multidimensional topic are considered rare. With regard to exploring key issues of the thematic intersection of geriatric glioblastoma patients, palliative care and neurosurgery using an approach that allows “interpretation and critique” [8] is particularly effective.

This literature review was performed using a semiautomated strategy. To capture publications on the primary population of older glioblastoma patients, we applied a search algorithm according to a rapid review design [9], while the content search for palliative care relevance was performed manually.

In July 2020, we performed a literature search using the following terms and trunks (*) in the electronic database PubMed (MEDLINE): (neurosurg*) AND (glioblastom*) AND ((geriatric*) OR (elder*) OR (old) OR (olde*)).

**Fig. 1:**
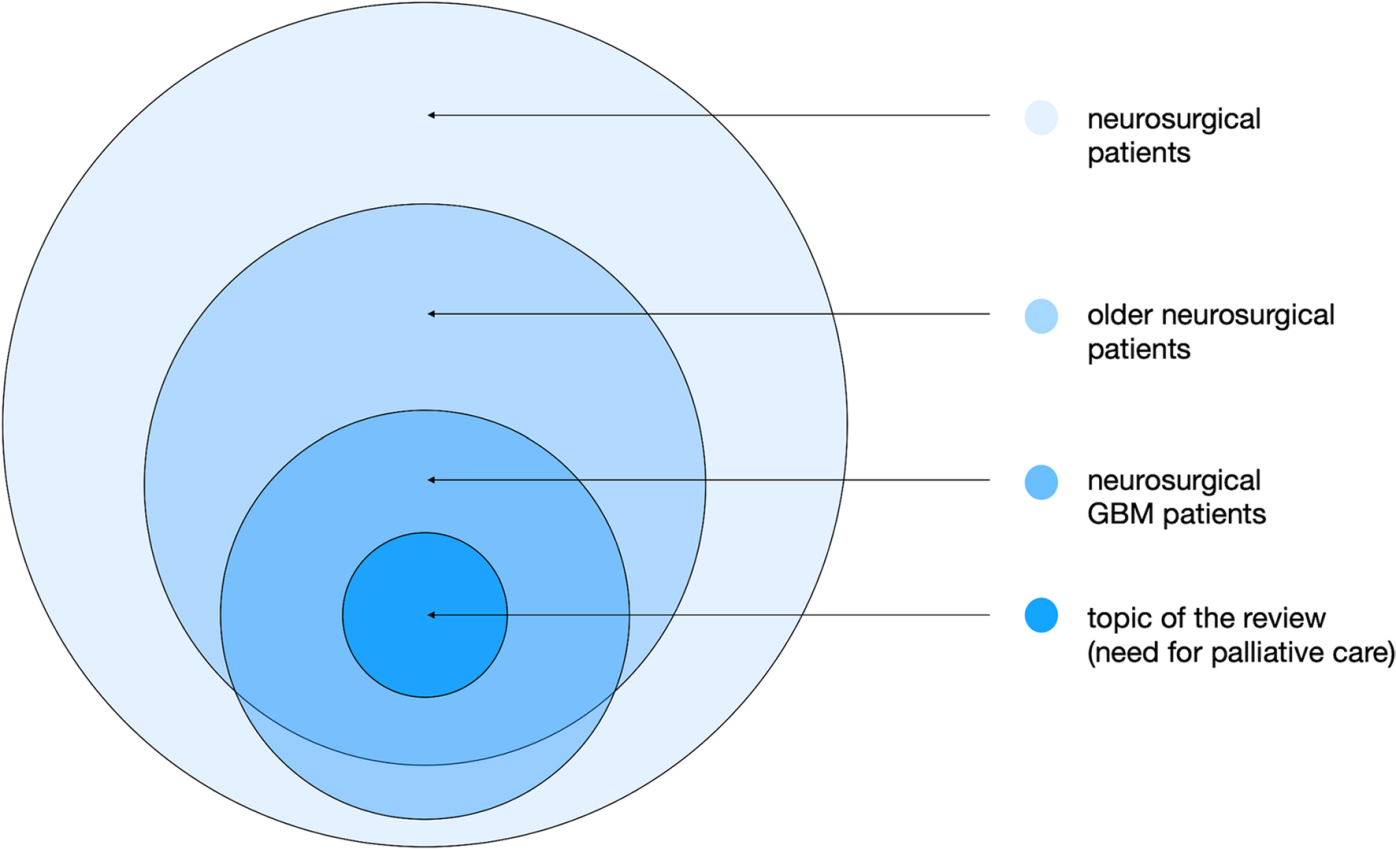
Schematic diagram of the thematic intersection

Two raters independently performed title/abstract screening. Studies were included for full text assessment if they reported on key issues of successful care practices and interdisciplinary cooperation at the intersection of palliative and neurosurgical care for older GBM patients (Fig. 1). All study types were considered for analysis. Basic research, pharmacological studies and articles presenting distinct surgical treatment options were excluded. To shed light on recent developments in interdisciplinary cooperation, only English articles published between August 2015 and August 2020 were considered in the search algorithm. After full text assessment crucial passages were identified in a qualitative consensus procedure.

## Results and discussion

The initial search yielded a total of 475 publications, of which 40 articles were full-text screened for relevance. Five articles were included in the current review after full-text assessment (n = 5), as summarised in Table 1. The subsequently conducted consensus procedure showed four categories, that correspond to the following key issues: patient-centeredness (need orientation and decision making), early palliative care, advance care planning, and multi-professionalism.

**Table 1:** Extracted findings in chronological order

Source	Study type	Extracted findings
Flanigan et al. [25]	Retrospective analysis	[…] age should be put into context of other negative prognostic factors when the decision between aggressive resection and more palliative care is being made
Halani et al. [26]	Literature review	[…] that age should not be a completely limiting factor when deciding which treatment options to pursue in elderly GBM patients […]
Ironside et al. [27]	Review	Newly diagnosed elderly patients (age 9 65–70 years) with glioblastoma should be treated with a patient-centered approach by a multi-disciplinary team Given the short life expectancy and the multiple complications related to the diagnosis and treatment of glioblastoma, discussions about advanced care planning (ACP) and palliative care should begin early in the trajectory of the patient’s disease There is some evidence that ACP can reduce hospital admissions and can improve the quality of life of patients with glioblastoma Early ACP conversations allow patients and their families to make timely decisions about their care by helping them to understand their treatment choices and define their goals of care In a disease with short expected survival, informed-decision making and frank discussions with patients and caregivers about treatment options, predicted treatment response and an emphasis on quality of life are of particular importance
Jordan et al. [28]	Literature review	[…] the treatment of glioblastoma in elderly patients requires an individualized approach for each patient Researchers have recognized the unique needs of this patient population […]
Pereira et al. [33]	Retrospective observational study	It is essential to consider other potential prognostic factors prior to surgery, to maximize the therapeutic effectiveness, and OS without compromising the patient’s quality of life, in an attempt to avoid unnecessary therapeutic aggression

### Patient-centeredness

#### Need orientation

According to the World Health Organization’s (WHO) definition, palliative care pursues a holistic approach with quality of life being the paramount therapeutic goal [1]. Since quality of life is subjectively conceptualised [10], the patient’s personal experience is attributed a particular significance (‘pain is what the patient says it is’). However, the central feature of palliative care is not the substitution of curing with palliation; rather, it is an inversion of priority. In that sense, priority is assigned to increasing and maintaining quality of life rather than to focusing on the usually very limited chances of recovery. It is therefore no longer somatic recovery (‘restitutio ad integrum’) that determines the further course of care and treatment but the ethical balance between a patient’s will and the medical indication, which must be constantly reviewed [11].

In this context, assessment of needs plays an important role at the end of life. Just as patients are not always aware of what they want (patient’s will), they are not always aware of what they need. Palliative care’s underlying philosophy shifts the focus from task-centered to person-centered care, acknowledging patients’ individual choices, desires and needs [12].

In neurosurgical patients, determination of needs and preferences becomes frequently challenging as the patients’ ability to express these through communication is sometimes limited or even impossible [13]. Additionally, this circumstance can lead to the social isolation of patients [14]. Although needs are highly diverse and unique to each patient, Sterckx et al. [15] summarised GBM patients’ needs into three thematic categories: Hope (including hope for quality of life), support (including the need for connection) and information (adapted to their vulnerability). It has been demonstrated, that these thematic categories coincide with the major themes for caregivers. However, it is important to understand the parallels as well as the differences of both perspectives. In any case, trained verbal and non-verbal communication is the most direct way to get an idea of the needs of patients and caregivers.

The problem of limited communication is especially true for very old patients, as they are often unable to adequately communicate [16] or hold themselves back because they do not want to be a burden on the staff [17]. Compared to younger patients, older patients may have different patterns of need and stress and complain less often about symptoms, especially pain, anxiety and nausea [18]. Particularly in the case of patients with cognitive limitations and dementia, there is a risk of underestimating the subjectively experienced burden [16]. Age-appropriate assessment tools, such as the BESD scale for assessing pain in dementia, can facilitate the recording of symptoms in everyday clinical practice and thus contribute to improving and maintaining quality of life [19].

#### Decision making

The literature indicates that there is an extensive debate about indications and suitability of certain therapeutic strategies depending on age. For this purpose, different algorithms, optimal treatment paradigms, treatment diagrams, patterns of care and predictors were identified, developed and evaluated [20–34]. It has been repeatedly demonstrated that there is a therapeutic dilemma that should not be solved at the expense of the patient’s quality of life [35]. Although GBM is associated with poor prognosis, especially in older patients [28], identified publications did not address that a patient-centered decision not only relies on medical indication but also on the individual will of the patient. It is therefore not surprising that 40% of high-grade glioma patients were not involved in the end-of-life decision making process at all [36]. An unspecified proportion of these may have been included too late in the decision-making process, because patients with GBM are prone to progressive cognitive impairments that interfere with their ability to make clinical decisions [37]. However, particularly in the context of end-of-life decision making, a tacit consent must not be supposed. Rather, the principle of self-determination in Western cultures makes explicit communication of decisions unavoidable. Shared decision making implies clinical knowledge of the treating physician, as well as subjective values and preferences of individual patients [38]. For this reason, shared decision making paves the way for value-based medicine and patient-centered care [39, 40].

Geriatric assessment (GA) [41] serves to properly prepare for indication-based recommendations. GA is a ‘multi-dimensional, multidisciplinary tool that can be used to evaluate medical, psychosocial, cognitive, and functional capabilities in older adults. The GA can identify previously undetected health conditions and predict treatment toxicities and overall survival in patients with cancer.’ [42] It has been shown to improve patient-centered and caregiver-centered communication about age-related concerns for older community patients with advanced cancer [41]. However, a complete decision-making process requires the integration of expectations, values and input from the patients themselves.

From a palliative care point of view, patient-centered decision making refers not only to the question of efficacy but also takes into account the question of whether a procedure makes sense. Under the heading of ‘medical futility’, the weighing of both poles is the subject of the debate on medical ethics, particularly with respect to the final stages of disease [43].

### Early palliative care

Despite optimal surgical and medical therapy, GBM remains almost invariably fatal. Assuming that palliative and life-prolonging treatment paths are mutually exclusive, involvement of palliative care services is often postponed until the last weeks or days of life. However, evidence-based consensus within the community of palliative care indicates that curative and palliative intervention should overlap in time [44–48], introducing the terms *early palliative care* or *early integration* into the pertinent literature [49]. Furthermore, this two-track approach also corresponds to the bifocal view of many patients as they simultaneously engage in the world and in the inner preparation for impending death, a process called *double awareness* [50].

Studies have shown that a whole range of outcomes have been improved, such as quality of life, survival, mood, caregiver burden, and reduction of aggressive treatment near the end of life [51].

The notion of *early integration* implies that palliative care for very older people should not begin in the last weeks of life (*terminal care*) but should be considered as soon as the first signs of deterioration and unmet needs occur. However, difficulty certainly arises when attempting to determine a fixed point of time for the integration of palliative care due to a large number of influencing factors, such as calendrical age, biological age, type and extent of multimorbidity, concurrent diseases and living conditions [52].

Neither age nor an assumed proximity to death should be decisive for the treatment path to pursue in older GBM patients [28]. Rather, integration of palliative care should depend on the phase of illness, and the intensity should be adapted according to symptom burden or the needs of the patients and their relatives [49, 53].

For this purpose, screening tools can be used that are not only geared to the success of treatment but also to query palliative care-related problems and needs. Instruments, such as the *Supportive and Palliative Care Indicators Tool* (SPICT), help to identify patients who would benefit from palliative care. Moreover, the SPICT has been successfully tested in a geriatric population [54]. General indicators of poor or deteriorating health acknowledged by this instrument include the following:

- Unplanned hospital admission(s)
- Poor or deteriorating performance status with limited reversibility (e.g., the patient stays in bed or in a chair for more than half the day)
- Dependence on others for care due to increasing physical and/or mental health problems
- The patient’s caregiver needs more help and support
- The patient has experienced significant weight loss over the last few months or remains underweight
- Persistent symptoms, despite optimal treatment of underlying condition(s)
- The patient (or family) asks for palliative care; chooses to reduce, stop or not have treatment; or wishes to focus on quality of life

Specific neurologically associated factors include the following:

- Progressive deterioration in physical and/or cognitive function, despite optimal therapy
- Speech problems with increasing difficulty communicating and/or progressive difficulty swallowing
- Recurrent aspiration pneumonia; breathless or respiratory failure
- Persistent paralysis after stroke with significant loss of function and ongoing disability

Studies investigating optimal timing to integrate palliative care for GBM patients are scarce [55]. At the moment, a randomised phase III clinical trial is being conducted to determine the efficacy of early specialised palliative care tailored to this patient population to improve quality of life [2]. In this study, shortly after diagnosis, patients are proactively and regularly contacted by the palliative care team (monthly contact by telephone, quarterly fixed face-to-face appointments).

Relevant case reports can be found in the literature on how the palliative care consultation services (PCCS) can be integrated into practice. Positive effects on the quality of dying have been demonstrated [56]. In contrast, Nehls and colleagues [57] illustrate the negative consequences of involving palliative expertise too late.

### Advance care planning

Early integration may make sense even before palliative care-related problems occur to provide advance care planning (ACP). The aim of ACP is to enable patients to plan ahead in terms of health care after carefully weighing personal values, goals and preferences. By accompanying the course of the illness in a process-oriented manner, supporting decision-making, attempting to standardise the documentation process and at the same time mediating among the patient, caregivers and practitioners, ACP goes beyond the scope of a living will. Thus, it is of particular importance in chronic progressive diseases with sometimes foreseeable impediments to the expression of will [58]. Ironside et al. [29] therefore argued that ACP …

- should begin early in the trajectory of the patient’s disease
- helps patients understand their treatment choices and define their goals of care
- can improve the quality of life of patients with glioblastoma
- can reduce hospital admissions

ACP is most often associated with palliative care in inpatient clinical settings. However, ACP plays an equally important role in the preceding and neighbouring care sectors, such as nursing homes and home settings. Especially with regard to (unplanned) hospital admissions out of home care settings, it was shown in a subgroup of GBM patients, that ACP led to significantly lower hospital readmission rates and intensive care unit utilization [59]. There is also evidence, which shows that ACP can reduce inappropriate hospital admissions of older patients (78 to 87 years) with cognitive impairment or dementia in nursing home settings [60]. Especially with regard to older adults, Frenchman et al. [61] demonstrated that an enhanced communication process is particularly important in this special patient population.

Although national strategies support the concept of ACP, in the reality of care, there is still a discrepancy between the demand for ACP and its actual implementation [62]. Experience shows that a successful implementation depends not only on the integration of palliative expertise but also on fruitful cooperation of all stakeholders involved.

### Multi-professionalism

To address the broadband needs of patients and their family members, the WHO definition of palliative care is characterised from a bio-psycho-socio-spiritual perspective. Physicians providing palliative medicine are thus only one of several equally important professions forming a multi-professional team that supports the patients and their caregivers [63]. In addition to qualified nurses and physicians, the palliative care team also includes other professions, such as psychologists, social workers, clergy, physiotherapists and arts therapists—keeping in mind that each profession contributes its individual competencies. For example, social workers are well trained in counseling and facilitating communication, which is a natural fit for implementing ACP [61]. Multi-professional skills are also particularly important in regard to understanding older adults in a holistic way. Thus, narrative medicine, performed by specialised professionals, can promote a systemic and integrated approach to treating older patients [64]. Accordingly, the European Association of Palliative Care (EAPC) designates the multi-professional approach as one of the core constituents of palliative care [65].

Alongside the classical field of medical responsibility, ‘soft skills’, such as compassion and mindfulness, are increasingly recognised to be of particular significance. Johnson [66] highlights the following key competencies in the palliative care of neurosurgical patients:

- (Empathic) Communication
- (Participatory) Decision making
- Support for psycho-socio-spiritual needs
- Pain management
- Treatment of nonpain symptoms
- Dealing with treatment complications
- Management of the dying phase
- Knowledge of palliative care structures

Engagement of neurosurgical experts and the palliative care team in an open and interdisciplinary exchange will result in beneficial effects, not only for patients and their family but also the entire care team. In hospitals, this interdisciplinary exchange can be ensured by the implementation of PCCS. PCCS offer palliative expertise to case-leading departments, leaving the final medical decision to the ward physicians who lead the case [67].

Geriatric palliative care is currently being established as an emerging “field of inter-specialty collaboration bringing together competences from geriatric medicine and palliative care to respond to the socio-demographic changes and challenges of older adults with severe and life-limiting conditions” [68]. As GBM predominantly occurs in older age, geriatric palliative care should engage in upcoming treatment and decision-making processes [69]. Thus, co-management of critical ill hospital patients between neurosurgery, geriatrics, and palliative care can increase use of hospital palliative care consultation, improve communication between teams and hospitalist confidence in their own palliative care skills [69]. Introduction of communication training by palliative care physicians, interprofessional rounds or interdisciplinary palliative boards serve to promote this concept.

Finally, multi-professionalism can be of considerable importance, since glioblastoma patients often undergo personality changes that are associated with distress and a reduced quality of life of patients and their informal caregivers [70]. There is evidence that three quarters of GBM patients require psychosocial intervention [71]. In particular, psychological interventions that support the course of treatment can help to improve patients’ emotional well-being and quality of life [72]. With regard to caregivers and relatives, support should not end with the death of the index person. Rather, the inclusion of bereavement counsellors should become a matter of routine, as caregivers may be heavily burdened beyond the death of the patient and are at risk of developing complicated grief [73]. This links palliative care not only to enhanced quality of end-of-life care but also to improved physical and mental health of the bereaved [74].

## Conclusions

This narrative literature review aimed to identify pioneering key issues of palliative care for older GBM patients to promote the integration of palliative care into neurosurgical practice. In this context, we found that four topics are currently being discussed within the literature: patient-centeredness (need orientation and decision making), early palliative care, advance care planning, and multi-professionalism. We highlighted benefits and barriers to the integration of palliative care in the context of these issues.

Beyond medical indication, knowledge about the (presumed) will of the patient is particularly relevant in palliative care. ACP may serve to establish a sustainable care and treatment concept guaranteed by the inclusion of a multi-professional palliative care team. The achievement of treatment goals is supported by multidimensional and continuous needs assessment. It is indispensable for palliative care teams to understand patients as individuals but also as part of a social network. Due to the potential complex disease history of older GBM patients, we have shown that this group is especially vulnerable and would therefore highly benefit from early integration of palliative care.

Furthermore, the review suggests that cross-sectoral thinking is an essential prerequisite for the successful care of older GBM patients and their families as they move through the various care settings, both in a forward-looking and sustainable manner. In this process, all involved health-care providers, organisations, and stakeholders must be aware of the need for ACP. In this context, palliative care may have a role of complementing the neurosurgical care of geriatric GBM patients and their relatives by providing expertise in addressing critical issues in the trajectories of incurable illnesses. Early integration may enable patients and their caregivers to adapt to the challenges they face within the time remaining. Palliative care involves acknowledgement of what cannot be changed—not as admission of failure but as a realistic option.

## Data Availability

Data sharing is not applicable to this article.
